# Investigation of γ-(2,3-Epoxypropoxy)propyltrimethoxy Silane Surface Modified Layered Double Hydroxides Improving UV Ageing Resistance of Asphalt

**DOI:** 10.3390/ma10010078

**Published:** 2017-01-19

**Authors:** Canlin Zhang, Jianying Yu, Lihui Xue, Yubin Sun

**Affiliations:** 1 State Key Laboratory of Silicate Materials for Architectures, Wuhan University of Technology, Wuhan 430070, China; zhangcanlin@whut.edu.cn; 2 Centrer for Materials Research and Analysis, Wuhan University of Technology, Wuhan 430070, China; sunyb0817@sina.com

**Keywords:** asphalt, layered double hydroxides, surface organic modification, ultraviolet ageing resistance, rheological properties, chemical compositions, morphology

## Abstract

γ-(2,3-Epoxypropoxy)propyltrimethoxy silane surface modified layered double hydroxides (KH560-LDHs) were prepared and used to improve the ultraviolet ageing resistance of asphalt. The results of X-ray photoelectron spectrometry (XPS) indicated that KH560 has been successfully grafted onto the surface of LDHs. The agglomeration of LDHs particles notably reduced after KH560 surface modification according to scanning electron microscopy (SEM), which implied that the KH560 surface modification was helpful to promote the dispersibility of LDHs in asphalt. Then, the influence of KH560-LDHs and LDHs on the physical and rheological properties of asphalt before and after UV ageing was thoroughly investigated. The storage stability test showed that the difference in softening point (Δ*S*) of LDHs modified asphalt decreased from 0.6 °C to 0.2 °C at an LDHs content of 1% after KH560 surface modification, and the tendency became more pronounced with the increase of LDH content, indicating that KH560 surface modification could improve the stability of LDHs in asphalt. After UV ageing, the viscous modulus (*G’’*) of asphalt significantly reduced, and correspondingly, the elastic modulus (*G’*) and rutting factor (G*/sin δ) rapidly increased. Moreover, the asphaltene increased and the amount of “bee-like” structures of the asphalt decreased. Compared with LDHs, KH560-LDHs obviously restrained performance deterioration of the asphalt, and helped to relieve the variation of the chemical compositions and morphology of asphalt, which suggested that the improvement of KH560-LDHs on UV ageing resistance of asphalt was superior to LDHs.

## 1. Introduction

Asphalt, which is the primary organic binding material, has been employed for several decades in road construction because of its outstanding viscoelastic properties [1,2]. However, like other organic compounds, asphalt is susceptible to ageing due to external environmental factors, such as sunlight, heat, and oxygen [3,4,5]. The ageing will lead to the degradation of the physical and rheological properties of asphalt, and eventually results in the deteriorated performance of the asphalt mixture (e.g., rutting, stripping, and cracking). Moreover, increasing traffic volumes and axle loads further accelerate the failure of asphalt [6]. Therefore, the improvement of the ageing resistance of asphalt is particularly important for the prolongation of the service life of asphalt pavement.

To improve the ageing resistance of asphalt, some methods have been attempted. Zargar et al. [7] noticed that the physical and rheological properties of asphalt after ageing were promoted by adding waste cooking oil. Feng et al. [8] investigated the effect of various ultraviolet absorbers on the ageing resistance of asphalt. The results showed that UV absorbents could improve the UV ageing resistance of asphalt, and the enhancement was largely determined by the kinds of UV absorbents and the nature of asphalt. However, the waste cooking oil and UV absorbents are organics, which are easily prone to ageing. Hence, the improvement of the ageing resistance of asphalt is limited. In some studies, inorganic materials have been used as anti-aging agents to improve the aging resistance of asphalt. Xiao et al. [9] discovered that carbon black was helpful in increasing the failure temperature of short-term aged asphalt binders and improving the rutting resistance at the high dosage; however, carbon black would seriously deteriorate the low-temperature performance of asphalt. Zhang et al. [10] disclosed that the addition of inorganic nanoparticles (nano-zinc oxide, nano-silica, and nano-titanium dioxide) was helpful in alleviating the performance deterioration of asphalt after UV ageing, but they would induce the photodegradation of asphalt simultaneously because of their photocatalytic activity [11].

**Figure 1:**
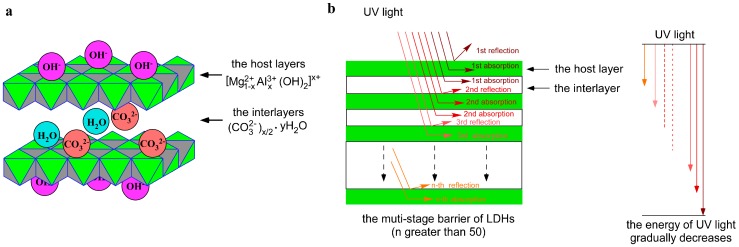
The structure model (**a**) and action mechanism of layered double hydroxides (LDHs) to impede UV light (**b**).

In recent years, layered double hydroxides (LDHs) have attracted considerable attention as ultraviolet light resistant materials. LDHs are a kind of anionic clay with layered crystal structures, whose general formula is [Mg^2+^_1−*x*_Al^3+^*_x_*(OH)_2_]*^x^*^+^(CO_3_^2−^)*_x_*_/2_ · yH_2_O; the structural model of LDHs is shown in Figure 1a [12,13]. Compared with general inorganic materials, such as spherical particles, LDHs have better ultraviolet resistance due to their multi-layered structure; their inorganic layer sheets can effectively shield ultraviolet light and the anions of interlamination and metal elements of the layer sheets can absorb ultraviolet light. The action mechanism of LDHs to impede UV light is depicted in Figure 1b [14,15,16,17]. Because of their excellent physical shielding and chemical absorption to ultraviolet light, LDHs have been applied for the UV ageing resistance of asphalt [12,14]. However, LDHs (strongly hydrophilic inorganic nonmetal powders) have difficulty in dispersing uniformly in the hydrophobic asphalt matrix. Moreover, LDHs modified asphalt is likely to have phase separation during storage and service. All these directly affect the sufficient utilization of the ultraviolet resistance of LDHs. Therefore, it is of great necessity to improve the compatibility between LDHs and asphalt. Recently, some solutions have been proposed; for example, anionic organic compounds (4,4′-stilbenedicarboxylic acid, sodium dodecylbenzenesulfonate, salicylic acid, etc.) were intercalated into the gallery space of LDHs to prepare organic LDHs by anion-exchange or re-construction [3,18]. However, the high charge density of LDHs layers leads to strong interlayer electrostatic interactions between the sheets, which makes LDHs surface organic modification difficult by these methods [19]. Simultaneously, due to the organic compounds immobilized in the interlayer space of LDHs, asphalt molecules could not enter the interlamination [20]. Hence, the reinforcing effect of these methods on the compatibility between LDHs and asphalt is limited. Silane coupling agents, often used to bridge organic and inorganic materials [21,22], have been employed to modify the organic surface of LDHs in some studies, and show effectiveness in improving the compatibility between LDHs and organics (e.g., polymethyl methacrylate, polystyrene, and epoxide resin) [23,24,25,26,27]. Based on this, silane coupling agents could be excellent modifiers to improve the dispersibility and stability of LDHs in hydrophobic asphalt matrix, and it has been reported in our previous studies that triethoxyvinylsilane (TEVS) surface organic LDHs could effectively improve the physical performances (penetration, softening point, ductility, etc.) of asphalt after UV ageing [28,29]. However, because TEVS contains a short organic molecular chain (vinyl), the improvement of TEVS surface modification for the compatibility between LDHs and asphalt is not remarkable. In order to make effective use of anti-ultraviolet LDHs, a silane coupling agent with a longer organic molecular chain would be more beneficial for the dispersibility and stability of LDHs in asphalt.

In this paper, LDHs organically modified by γ-(2,3-epoxypropoxy)propyltrimethoxy silane (KH560) were used to improve the UV ageing resistance of asphalt. The influence of surface organic LDHs on the rheological properties, chemical compositions, and morphology of asphalt before and after UV ageing were thoroughly investigated by dynamic shear rheometer (DSR) tests, thin-layer chromatography with flame ionization detection (TLC-FID), and atomic force microscopy (AFM), respectively.

## 2. Experimental Section

### 2.1. Materials

**Figure 2:**
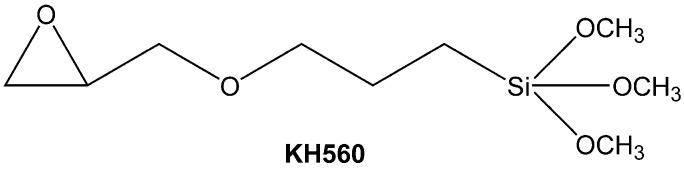
Chemical structure of γ-(2,3-epoxypropoxy)propyltrimethoxy silane (KH560).

Virgin asphalt (SK-70) was offered by SK Corp., Ulsan, Korea. The chemical compositions and physical properties of virgin asphalt are presented in Table 1. LDHs were supplied from Beijing Tech-layer Corp., Ltd., Beijing, China. KH560 (analytically pure) was provided by Sinopharm Chemical Reagent Co., Ltd., Shanghai, China. The chemical structure of KH560 is exhibited in Figure 2.

### 2.2. Synthesis of KH560 Surface Modified Layered Double Hydroxides

First, LDHs powders (30 g) were pre-dispersed in an ethanol-water mixture (5%, 300 mL) at 50 °C for 0.5 h, and KH560 (1 mL) was dissolved in an ethanol-water mixture (90%, 50 mL) under vigorous stirring. Then, the LDH slurry and KH560 solution were mixed under continuous stirring, and the pH of the mixed solution was adjusted to 3.5 by adding acetic acid dropwise. After that, the mixture was kept at 50 °C for 3 h with vigorous stirring in an atmosphere of N_2_. Subsequently, the mixtures continued to react at 70 °C under N_2_ protection for about 0.5 h. Finally, the reaction product (white precipitate) was separated and repeatedly washed with deionized water (six times), and then dried at 80 °C for 24 h in a vacuum drying oven. The modified LDHs were ground into powder with a size of 0.075 mm, and KH560 surface organic LDHs were denoted as KH560-LDHs for simplicity in later parts.

### 2.3. Preparation of KH560-LDHs Modified Asphalt

The preparation of modified asphalts was performed using melt blending. Firstly, the virgin asphalt was heated to be fluid with an oil bath treatment at 140 ± 5 °C in an iron container, and the appropriate content of KH560-LDHs and LDHs were added into the melting binders. To make LDH particles disperse homogenously in binders, the mixture was blended at a shearing speed of 4000 rpm for 1.5 h under the condition of 170 ± 5 °C. Then, modified binders were preserved for later related experiments and tests. The virgin asphalt underwent the same preparation process to keep the experimental consistency for all samples. Additionally, for convenience, virgin asphalt, LDH modified asphalt, and KH560-LDHmodified asphalt were abbreviated to VA, LMA, and KH560-LMA, respectively.

### 2.4. UV Ageing Procedures

The UV accelerated ageing of asphalt in the laboratory was divided into two steps. Firstly, the rolling thin film oven test (RTFOT) based on ASTM D1754 was carried out on all the asphalt samples (VA, LMA, and KH560-LMA), which was applied to simulate the thermo-oxidative ageing of binder that occurs during the mixing and paving of the asphalt mixture. Secondly, the residual products from RTFOT were poured into circular pans (diameter: 140 ± 0.5 mm, thickness: 3.2 mm), and then the circular pans were placed in a UV accelerated weatherometer (ST101F-2AB, Zhongke Equipment Co., Ltd, Wuhan, China) for 7 days, which was used to imitate the photo-oxidation ageing of asphalt during the service process. The intensity of the UV radiation and ageing temperature were set at 2000 μW/cm^2^ and 60 ± 3 °C, respectively.

### 2.5. Characterization

X-ray photoelectron spectroscopy (XPS, ESCALAB 250Xi, Thermo Fisher Scientific Inc., Wellesley, MA, USA) was utilized to measure the elemental composition and chemical state of LDHs before and after KH560 surface organic modification. The measurement was executed with an Al Kα X-ray source (1486.6 eV) and a vacuum condition of 1.0 × 10^−9^ Pa.

A field emission scanning electron microscope (FE-SEM, Zeiss Ultra Plus, Oberkochen, Germany) was adopted to observe the morphology of LDHs and KH560-LDHs, and the operating voltage of the microscope was 5 kV.

The morphology of binder samples was investigated by atomic force microscopy (Model DI Nanoscope IV, Veeco Company, New York, NY, USA). The test was performed under tapping mode, and the observation size of each sample was a square area of 12 × 12 μm.

### 2.6. Storage Stability Test

The storage stability test was used to investigate the dispersion and stability of LDHs or KH560-LDHs in asphalt in this paper. The test procedure was as follow: Asphalt samples were poured into aluminum tubes with a diameter of 32 mm and a height of 160 mm. After that, the aluminum tubes were sealed and kept vertically in an oven at 163 °C for 48 h, and the tubes were cut into three equal parts when cooled to ambient temperature. Finally, the top and bottom parts were subjected to the ring and ball (R&B) softening point test, and the effect of the KH560 surface organic modification on the dispersion and stability of LDHs particles in the asphalt can be investigated by comparing the difference value of softening points (Δ*S*) between the two parts. That is, the smaller the Δ*S*, the better the dispersion and stability.

### 2.7. Dynamic Shear Rheometer Test

Dynamic rheological properties of all asphalt samples (VA, LMA, and KH560-LMA) were achieved from dynamic shear rheometer (DSR, MCR101, Anton Paar Corp., Graz, Australia). Asphalt samples were heated until they were sufficiently fluid, and then the hot binders were poured into a mold and cooled to room temperature. The asphalt disk was removed from the mold and placed on the lower plate of the DSR device. The lower plate was heated to make the binders soft. After that, the gap between the top and bottom plates was gradually reduced to the target value. The temperature sweep test was operated under the strain-controlled mode with a heating rate of 2 °C/min and a constant frequency of 10 rad/s. The parallel plates with a diameter of 8 mm were applied during the low-temperature regions (ranging from −10 °C to 30 °C) and the gap between the top and bottom plates was 2 mm. Correspondingly, the diameter was 25 mm during the high-temperature regions (from 30 °C to 80 °C) and the gap was 1 mm.

### 2.8. Thin-Layer Chromatography with Flame Ionization Detection Test

**Figure 3:**
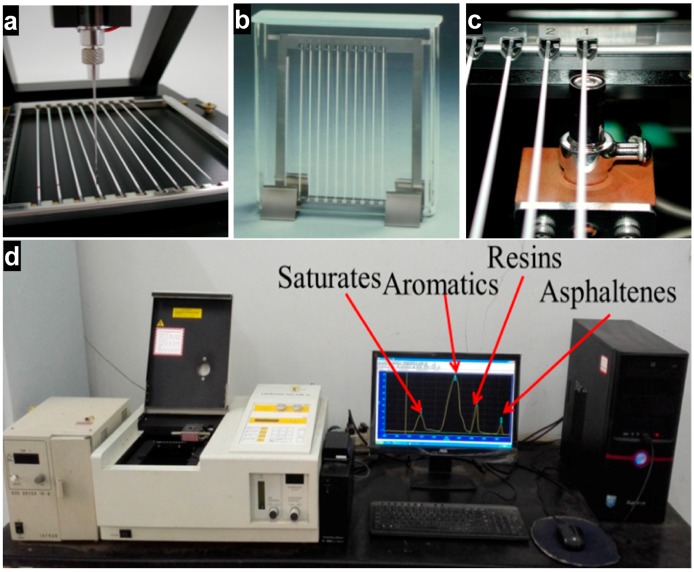
Thin-layer chromatography with flame ionization detection (TLC-FID) test: (**a**) spotting; (**b**) separating; (**c**) ignition; (**d**) analysing.

First, all samples (VA, LMA, and KH560-LMA) were dissolved in methylene chloride with a concentration of 2 g/L. Then, asphalt solutions (about 2 μL) were spotted on ten chromatographic columns. In order to separate the chemical components of asphalt, the chromatographic columns were successively fractionated in the three different solvents (*n*-heptane, toluene/*n*-heptane, and toluene/ethanol). The experimental procedure is shown in Figure 3. Finally, the chromatographic columns were analyzed by TLC-FID (Iatron Laboratories Inc., Tokyo, Japan). Ten groups of data were obtained from each sample, and the test result was acquired from the analysis of SIC-480 II.

## 3. Results and Discussion

### 3.1. Characterization of KH560-LDHs

#### 3.1.1. X-ray Photoelectron Spectroscopy

**Figure 4:**
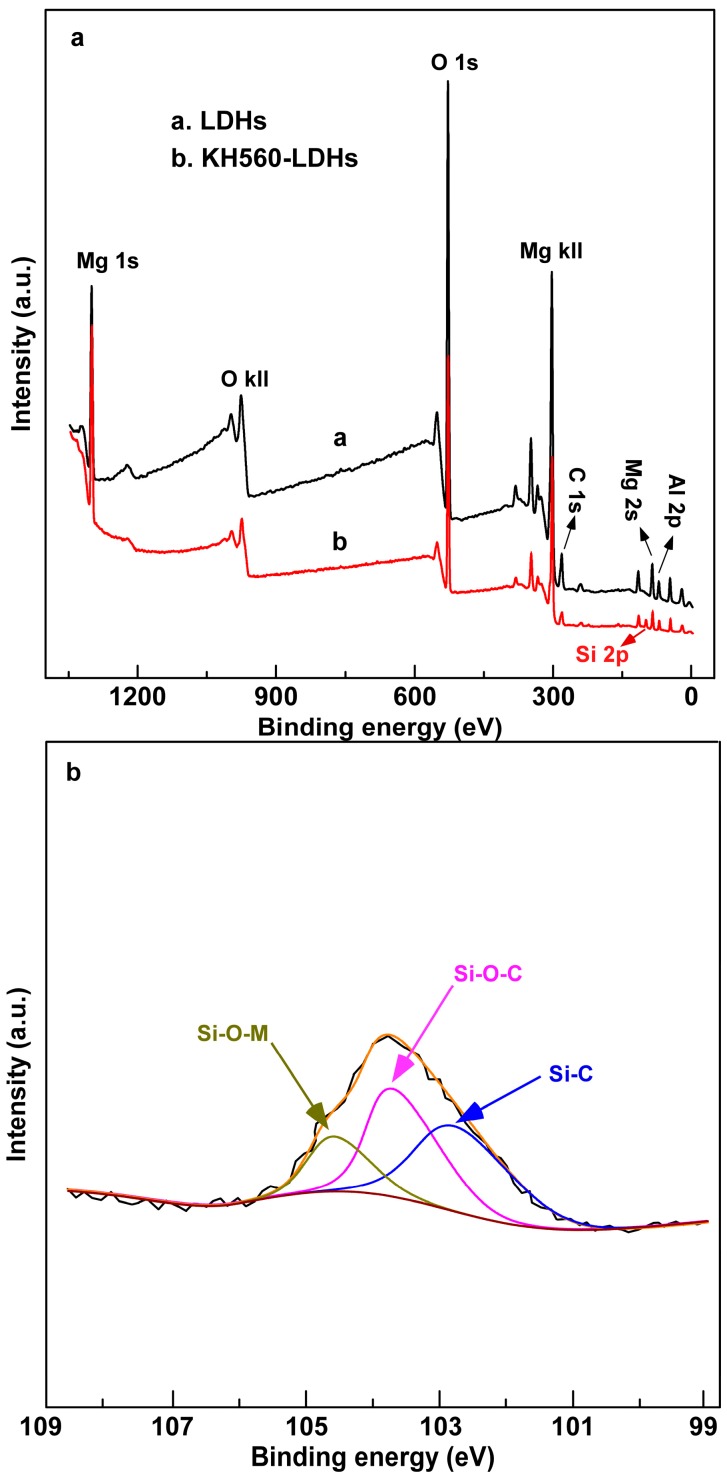
X-ray photoelectron spectroscopy (XPS) spectra of : (**a**) LDHs (black line) and KH560-LDHs (red line); (**b**) Si 2p core region.

The XPS spectra of LDHs before and after KH560 surface organic modification are portrayed in Figure 4. In the wide scan survey spectra (Figure 4a), the binding energy at 74.5 eV for Al 2p, 88.6 eV for Mg 2s, and 1303.9 eV for Mg 1s are assigned to aluminum hydroxide Al(OH)_m_ and magnesium hydroxide Mg(OH)_n_ in the LDHs, respectively. Compared with LDHs, a new binding energy at 103.8 eV for Si 2p can be observed in the spectra of KH560-LDHs, which suggests the presence of the Si element in KH560-LDHs. Therefore, a preliminary conclusion could be deduced, that is, KH560 has been grafted onto the surface of LDHs. The high energy resolution spectra for Si 2p is plotted in Figure 4b; the peaks located at 102.5 eV, 103.8 eV, and 104.2 eV are the binding energies of Si–C, Si–O–C, and Si–O–M (M = Mg or Al), respectively [25]. Among them, the Si–C and Si–O–C bonds derive from the KH560 molecules, and the formation of Si–O–M is from the reaction between Si–OH and the hydroxyl groups on the surface of LDHs [25,30]. The results of the XPS spectra of LDHs and KH560-LDHs indicate that KH560 molecules have been well bound to the surface of LDHs by covalent bonds.

#### 3.1.2. Scanning Electron Microscopy

**Figure 5:**
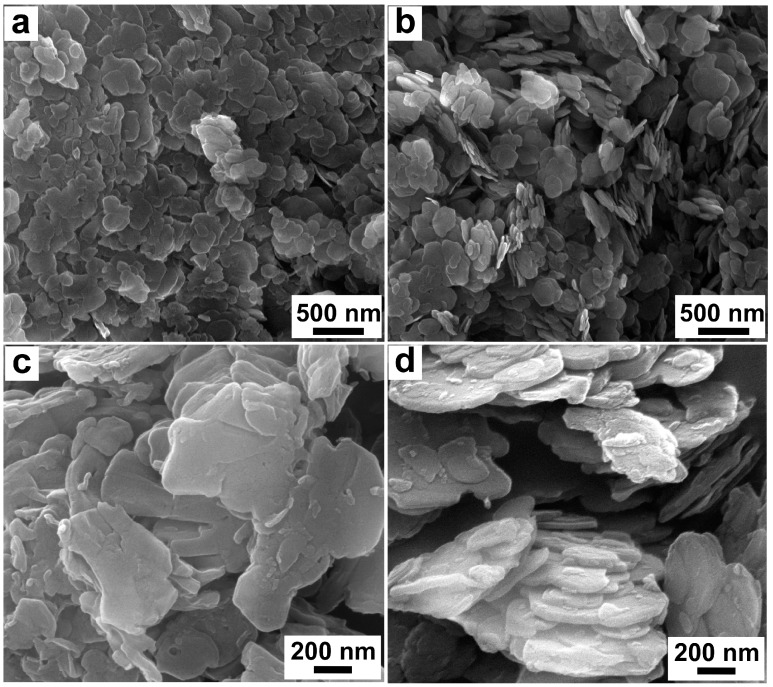
Scanning Electron Micoscopy (SEM) micrographs of LDHs (**a**,**b**) and KH560-LDHs (**c**,**d**).

The micro-morphology of LDHs and KH560-LDHs is shown in Figure 5. It can be seen that all the samples have a typical lamellar structure with irregular shape. In the SEM image of LDHs (Figure 5a,c), many LDHs particles tend to adhere together to form large-scale ones, which may be attributed to a large amount of hydroxyl groups (polar group) distributed on the surface of LDHs [28,31]. However, the agglomeration state between LDHs particles noticeably reduces after KH560 surface modification (Figure 5b,d), which may contribute to the decrease of hydroxyl groups on the surface of LDHs after KH560 grafted onto it. In addition, the interstice between KH560-LDHs is significantly larger than that of LDHs, which is a benefit towards enhancing the dispersibility of LDHs in asphalt.

### 3.2. Storage Stability of Modified Asphalt

**Figure 6:**
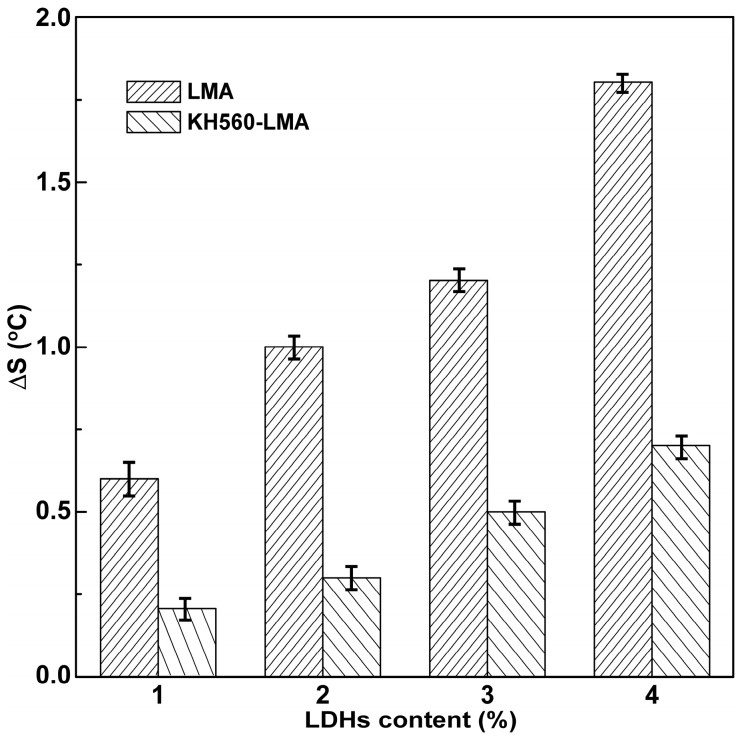
The storage stability of LDHs modified asphalt (LMA) and KH560-LMA.

Due to the phase separation that easily occurs between LDHs and asphalt during storage, the stability of LDHs in asphalt is of considerable importance, which has a direct impact on whether the ultraviolet resistance of LDHs can be effectively utilized. In order to make LDHs and KH560-LDHs disperse homogenously in asphalt, the mixture was blended at a high shearing speed (4000 rpm) during the preparation process. The storage stability of LMA and KH560-LMA is plotted in Figure 6. The difference in the softening point between the top and the bottom (Δ*S*) of the LMA increases rapidly with the increase of the LDHs content. The Δ*S* of LMA is 0.6 °C at an LDHs content of 1%, and the Δ*S* increases to 1.8 °C when the content reaches 4%. However, the increasing rate of Δ*S* obviously slows down after LDHs modified by KH560; the Δ*S* of KH560-LMA increases from 0.2 °C to 0.7 °C as the content increases from 1% to 4%. The result indicates that KH560 surface organic modification shows effectiveness in improving the stability of LDHs in the hydrophobic asphalt matrix, which is caused by the surface transformation of LDHs from hydrophilic to hydrophobic after KH560 surface organic modification.

### 3.3. Dynamic Viscoelastic Properties

#### 3.3.1. Low Temperature Sweep

**Figure 7:**
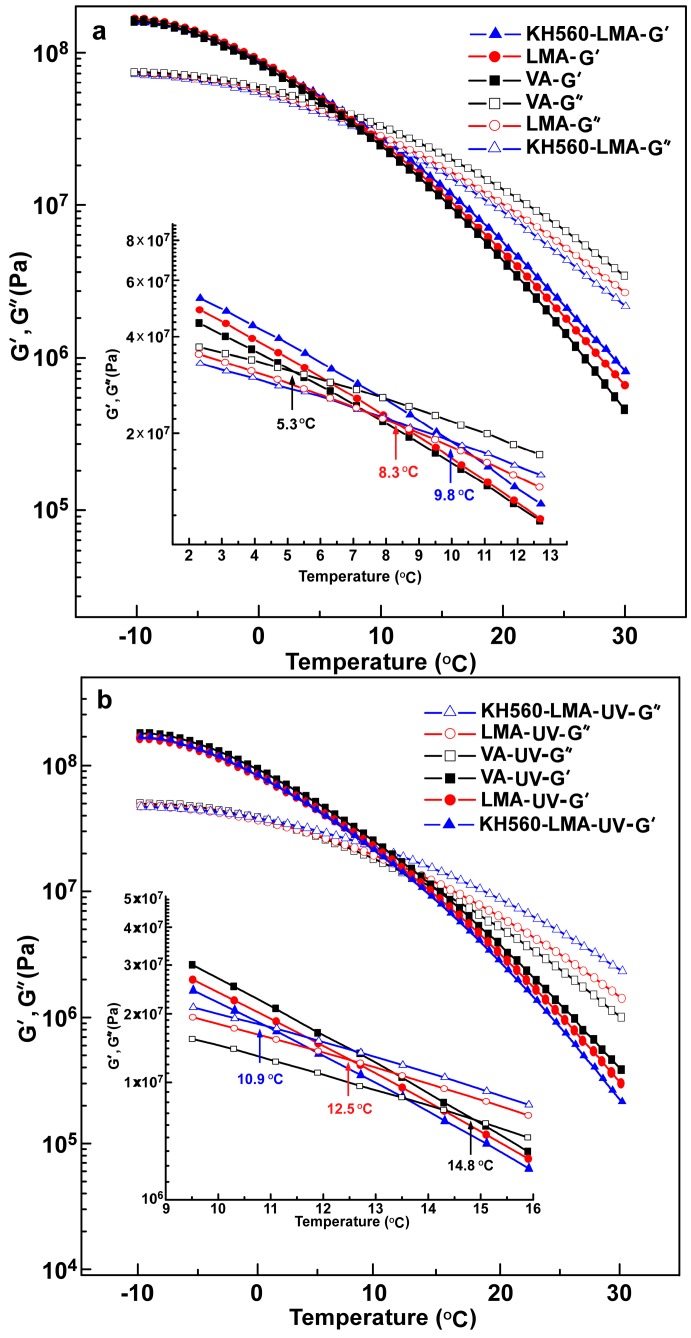
Viscous modulus (*G’’*) and elastic modulus (*G’*) vs. temperature for all asphalt samples: (**a**) before and (**b**) after UV ageing.

As a viscoelastic material, asphalt exhibits viscous behavior, elastic behavior, or both at different temperature ranges [9], which is of significance for the extensive application of the asphalt mixture. The viscous modulus (*G’’*) and elastic modulus (*G’*) of LMA and KH560-LMA at low temperatures (from −10 °C to 30 °C) before and after UV ageing are depicted in Figure 7. It can be observed that the *G’’* and *G’* of all binders decrease with the increase of temperature and the descending rate of *G’* is faster than that of *G’’*. Interestingly, there is an intersection point between *G’’* and *G’* for each asphalt sample, and the intersection point corresponds to a temperature (denoted as *T_p_*). The *G’* value is greater than the *G’’* value before *T_p_*, whereas the magnitude of *G’’* and *G’* reverses after *T_p_*. In other words, asphalt samples show more elastic property before the *T_p_*, and exhibit more viscous property when the *T_p_* is exceeded, as exhibited in Figure 7a and Table 2. The *T_p_* of VA, LMA, and KH560-LMA are 5.3 °C, 8.3 °C, and 9.8 °C, respectively. The *T_p_* of asphalt becomes larger with the addition of LDHs and KH560-LDHs, which indicates that LDHs or KH560-LDHs enhance the stiffness of asphalt.

The *G’’* and *G’* values of asphalt samples at low temperatures after UV ageing are displayed in Figure 7b and Table 2. It can be seen clearly that the *G’’* of all samples decreases in varying degrees after UV ageing, while *G’* increases. The *T_p_* (the intersection between *G’’* and *G’*) of VA, LMA, and KH560-LMA are 14.8 °C, 12.5 °C, and 10.9 °C, respectively, and the *T_p_* increments are 9.5 °C, 4.2 °C, and 1.1 °C in comparison to the asphalt samples before UV ageing. The results indicate that asphalt becomes hard and brittle after UV ageing, which will lead to the cracking of the asphalt mixture and shortens the service life. However, it is worth mentioning that the performance deterioration of asphalts caused by UV is evidently restrained with the introduction of LDHs and KH560-LDHs. Moreover, KH560-LDHs modified asphalt shows better anti-ultraviolet ageing compared with LDHs modified asphalt, which is due to the better dispersibility and stability of KH560-LDHs in the hydrophobic asphalt matrix.

#### 3.3.2. High Temperature Sweep

**Figure 8:**
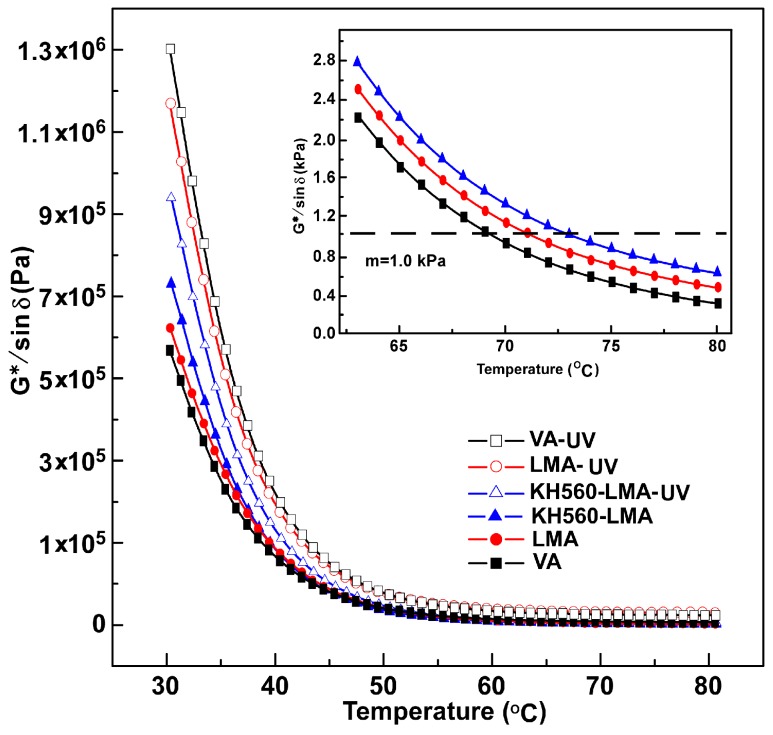
Temperature dependence of G*/sin δ of all binders before and after UV ageing.

Rutting factor (G*/sin δ) is the high temperature performance parameter of asphalt, which is applied to measure the rutting resistance of asphalt in the high temperature region [32]. The larger the rutting factor, the better high temperature rutting resistance of the asphalt. The G*/sin δ of all asphalts (ranging from 30 °C to 80 °C) before and after UV ageing are presented in Figure 8. The rutting factors of LMA and KH560-LMA are slightly improved compared with VA, which implies that LDHs or KH560-LDHs could enhance the rutting resistance of the binder. Furthermore, according to the Strategic Highway Research Program (SHRP), the critical temperature of G*/sin δ (T_G*/sin__δ_) at 1 kPa is for fresh asphalt, which is employed to estimate the high temperature performance grade of asphalt [29,32]. The T_G*/sin__δ_ of all asphalt samples are listed in Table 3. The T_G*/sin__δ_ of VA, LMA, and KH560-LMA are 69.5 °C, 71.5 °C, and 72.9 °C, respectively. The high temperature performance grade of virgin asphalt is promoted from PG 64 to PG 70 with the introduction of KH560-LDHs and LDHs, which further verifies the enhancement of KH560-LDHs and LDHs on the rutting resistance of the binder in the high temperature region.

After UV ageing, the rutting factors of all asphalt samples increase significantly. However, the increased amplitude of rutting factors for virgin asphalt and modified asphalts are different; the increase of VA is the most significant, followed by LMA, and KH56-LMA has the smallest increase. As mentioned earlier, asphalt becomes stiff and brittle after UV ageing, which will rapidly increase the G*/sin δ of the asphalt. Although the G*/sin δ of VA is higher than that of LMA and KH560-LMA after UV ageing, it reflects that VA is vulnerable to ageing rather than having better rutting resistance in the accelerated ageing process. The results imply that LDHs particles could inhibit the hardening of asphalt in the UV ageing process, especially for KH560-LDHs.

### 3.4. SARA Analysis

Asphalt is generally divided into four chemical components, that is, saturate (Sa.), aromatic (Ar.), resin (Re.), and asphaltene (As.) [33,34]. The chemical components of asphalt will change during UV ageing. Therefore, the variation of the chemical components of the binders before and after UV ageing can be employed to evaluate the UV ageing resistance of the asphalt. The Colloid stable index (*C_I_*, the ratio of continuous phase and dispersed phase) and the Gelation index (*G_I_*, the larger the *G_I_*, the more serious the gelation and ageing degree) are frequently used to measure the change of chemical components of binders. The *C_I_* and *G_I_* are calculated as Equations (1) and (2), respectively.

*C_I_* = (Aromatic + Resin)/(Saturate + Asphaltene) (1)

*G_I_* = (*C_I_* before UV ageing − *C_I_* after UV ageing)/(*C_I_* before UV ageing) (2)

The chemical components of all binders before and after UV ageing are shown in Table 4. It can be seen that the four chemical components of LMA and KH560-LMA are approximately consistent with that of VA before UV ageing, and the *C_I_* value of VA, LMA, and KH560-LMA are quite close (3.425, 3.429, and 3.436, respectively), which indicates that the addition of LDHs or KH560-LDHs has no effect on the chemical components and colloidal stability of the asphalt. After UV ageing, the aromatic content noticeably decreases, and correspondingly, the asphaltene content dramatically increases, and the contents of saturate and resin of VA change slightly. However, the variation of the chemical components of LMA and KH560-LMA are obviously relieved compared with VA. The *C_I_* values of the three asphalt samples are successively 1.934, 2.561, and 3.063, and the corresponding *G_I_* values are 43.5%, 25.3%, and 10.9%. The results suggest that the gelation of asphalt significantly increases after UV ageing, and that LDHs and KH560-LDHs can alleviate this variation of asphalt, particularly of KH560-LDHs.

Due to the UV light with high energy, some chemical bonds of asphalt would be easily broken to form free radicals during the UV ageing process. The free radical is very unstable when exposed to oxygen [20]. The oxidation reaction of asphalt contributes to the rapid decrease in the aromatic content and to a significant increase in asphaltene (the highest polar among the four chemical components), that is, the polarity of asphalt increases after UV ageing. Moreover, the increase of asphaltene would enhance the stiffness of asphalt [35], which is consistent with the result of the rheological properties of asphalt. The addition of LDHs and KH560-LDHs effectively mitigates the chemical conversion of asphalt during the UV ageing process. The multilayer laminar structures of LDHs could physically shield and chemically absorb UV light [28], meanwhile, also preventing the infiltration of oxygen into the asphalt to some extent. Because of the better dispersibility and stability, the improvement of KH560-LDHs on the UV ageing resistance of asphalt is superior to LDHs.

### 3.5. Atomic Force Microscopy Analysis

**Figure 9:**
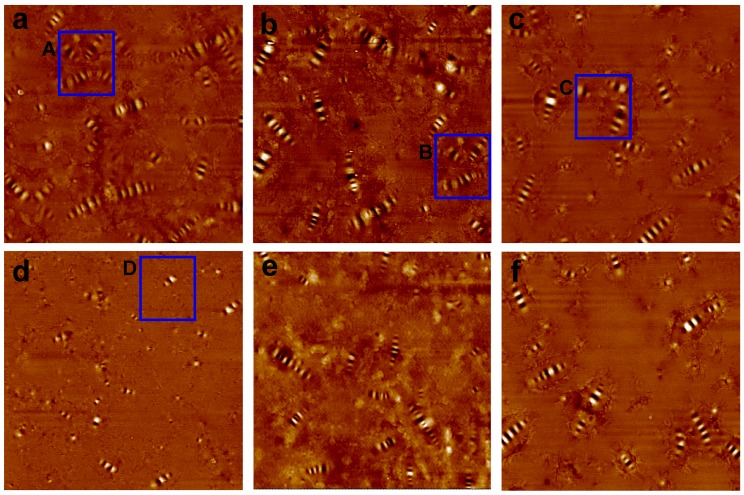
Topographic atomic force microscopy (AFM) images of (**a**,**d**) VA before and after UV ageing; (**b**,**e**) LMA before and after UV ageing and (**c**,**f**) KH560-LMA before and after UV ageing.

Microstructures of VA, LMA, and KH560-LMA are portrayed in Figure 9. It can be observed clearly that some black-and-white parallel stripes (called “bee-like” structures) appear in topographic images of all asphalt samples. On the basis of previous studies, the “bee-like” structure is attributed to the co-crystallization of asphaltenes and microcrystalline waxes (>C_40_) at the test temperature (about 5 °C) [3,36,37]. As displayed in Figure 9a, the “bee-like” structures are apt to flock together (as in the A region in Figure 9a), which is related to the polar and hydrogen bonding of the “bee-like” structure. However, the aggregation between the “bee-like” structures is inhibited after adding LDHs and KH560-LDHs (such as in the B and C regions in Figure 9a,b), which is due to the layer structures of LDHs hindering the movement of asphalt molecules during the cooling process (from flowing to solid). Furthermore, the inhibitory effect of KH560-LDHs is better than that of LDHs.

After UV ageing, the amount of “bee-like” structures in VA noticeably decreases. In addition, it is noteworthy that the size of the “bee-like” structures becomes smaller, and the contour becomes blurred (as in the D region in Figure 9d). These changes in the microstructures of asphalt are closely related to the increasing polarity of the molecular asphalt during the UV ageing [3,36]. The generation of polar groups such as carboxyl, hydroperoxide, ketonic, and acidic groups [3,38], decreases the agglomeration of asphaltenes, and on the other hand, blocks the co-crystallization of the microcrystalline waxes and asphaltenes, which finally induces the disappearance and diminution of the “bee-like” structures after UV ageing. However, it is noteworthy that the alteration of the microstructure of modified asphalt is obviously mitigated compared with VA. Moreover, the variation of the microstructure of KH560-LMA is the least amongst all the asphalt specimens after UV ageing. The result implies that LDHs can block the UV ageing of asphalt and the effect is better after KH560 surface organic modification.

## 4. Conclusions

LDHs surface modified by KH560 were prepared and used to alleviate the performance deterioration of asphalt after UV ageing. The structures of LDHs before and after surface modification were characterized by XPS and SEM. Simultaneously, the influence of LDHs and KH560-LDHs on the rheological properties, chemical compositions, and microstructures of asphalt before and after UV ageing was investigated thoroughly. Based on the analysis of the experimental results, the conclusions can be summarized as following:

1. According to XPS, KH560 has been successfully grafted onto the surface of LDHs, which was conducive to improving the stability of LDHs in the hydrophobic asphalt matrix. The SEM results showed that the agglomeration between LDHs particles was significantly weakened after KH560 surface modification, indicating that the dispersibility of LDHs in asphalt could be promoted by KH560 surface modification.
2. The Δ*S* of KH560-LMA was much less than that of LMA, and the superiority of KH560-LMA became more prominent as the content of LDHs increased. The addition of KH560-LDHs could improve the high-temperature rutting resistance of asphalt.
3. After UV ageing, the *G’’* of asphalt significantly reduced, and correspondingly, the *G’’* and G*/sin δ rapidly increased. The reduction of *G’’* of the asphalt and the increase of *G’* and G*/sin δ were inhibited after adding LDHs particles. Moreover, KH560-LDHs showed more effectiveness in hindering the rheological performance deterioration of asphalt in comparison to LDHs.
4. The UV ageing of asphalt resulted in the significant reduction of aromatic content and the increase of asphaltene, which was unfavorable to the colloidal stability of asphalt and shortened the service life of asphalt pavement. Compared with LDHs, KH560-LDHs could better alleviate the variation of chemical compositions, implying that KH560-LDHs were superior to LDHs in improving the UV ageing resistance of asphalt.
5. The amount and dimension of “bee-like” structures of asphalt noticeably decreased after UV ageing, which was due to the increasing polarity of asphalt. The change of “bee-like” structures of asphalt was mitigated noticeably with the introduction of KH560-LDHs, which further indicated that KH560 surface organic modification contributed significantly in improving the UV ageing resistance of LDHs modified asphalt.

## Figures and Tables

**Table 1:** Chemical compositions and physical properties of virgin asphalt.

	Items	Measured Values
Chemical constituents	Asphaltenes (%)	9.7
	Saturates (%)	13.3
	Resins (%)	31.3
	Aromatics (%)	45.7
Physical properties	Penetration (25 °C, 0.1 mm)	73
	Ductility (15 °C/10 °C, cm)	>150/16.5
	Softening point (°C)	48.8
	Viscosity (135 °C, Pa·s)	0.49

**Table 2:** The *T_p_* of all asphalt samples before and after UV ageing.

Asphalt Samples	Before UV Ageing (°C)	After UV Ageing (°C)	Increase of *T_p_* (°C)
VA	5.3	14.8	9.5
LMA	8.3	12.5	4.2
KH560-LMA	9.8	10.9	1.1

VA, virgin asphalt; LMA, LDHs modified asphalt; KH560-LMA, KH560-LDHs modified asphalt.

**Table 3:** Critical temperature of fresh binders.

Asphalt Samples	VA	LMA	KH560-LMA
T_G*/sin__δ_ (1 kPa at 10 rad/s)	69.5 °C	71.5 °C	72.9 °C
Performance grade	PG 64	PG 70	PG 70

**Table 4:** Chemical compositions of all asphalt specimens before and after UV ageing.

Asphalt Samples	Fresh					Aged					*G_I_* (%)
	Sa. (%)	Ar. (%)	Re. (%)	As. (%)	*C_I_*	Sa. (%)	Ar. (%)	Re. (%)	As. (%)	*C_I_*	
VA	12.86	45.92	31.48	9.74	3.425	11.93	33.59	32.33	22.15	1.934	43.5
LMA	12.97	45.85	31.57	9.61	3.429	12.14	39.38	32.54	15.94	2.561	25.3
KH560-LMA	12.88	45.35	32.11	9.66	3.436	12.01	41.54	33.85	12.6	3.063	10.9

Sa., saturate; Ar., aromatic; Re., resin; As., asphaltene.
